# A Method for Characterizing the Chemical Heterogeneity of Comb-Copolymers and Its Dependence on Synthesis Routes

**DOI:** 10.3390/polym13121921

**Published:** 2021-06-09

**Authors:** Stefanie Anne Weckwerth, Wolfgang Radke, Robert J. Flatt

**Affiliations:** 1 Institute for Building Materials, ETH, CH-8093 Zürich, Switzerland; weckwers@ethz.ch; 2 Polymer Standards Service GmbH, DE-55120 Mainz, Germany; wradke@pss-polymer.com

**Keywords:** size exclusion chromatography, dual concentration detection, poly(carboxylate ether), superplasticizers, comb copolymers, chemical dispersity, molecular heterogeneity

## Abstract

The heterogeneity in chemical structure of polymers is difficult to characterize and consequently remains an often-overlooked factor in mechanistic studies of functional polymers, as well as in their industrial scale optimization. In this study, we present a method to characterize chemical heterogeneity and apply it to illustrate how it can be affected differently in different synthesis routes. The polymers used are comb-copolymer dispersants used in particulate suspensions which are composed of a polycarboxylate backbone onto which PEG side chains are grafted. The largest use of these polymers concerns concrete, where they are referred to as poly(carboxylate ether) (PCE) superplasticizers and produced at a very large industrial scale. Apart from their practical relevance, PCEs provide a good test case for studying the means and benefits of characterizing chemical heterogeneity. Indeed, the simple addition of a UV detector to a traditional SEC setup with RI detection allowed us to monitor variations in the grafting ratio in dependence on the molecular size. We show that the synthesis pathway significantly impacts the chemical heterogeneity. The suggested method is versatile and can be adapted for a wide range of hydrophilic copolymers. Thus, we present a tool to comprehensively analyze the molecular heterogeneity of dispersants and give a deep insight into their chemical dispersity.

## 1. Introduction

**Figure 1:**
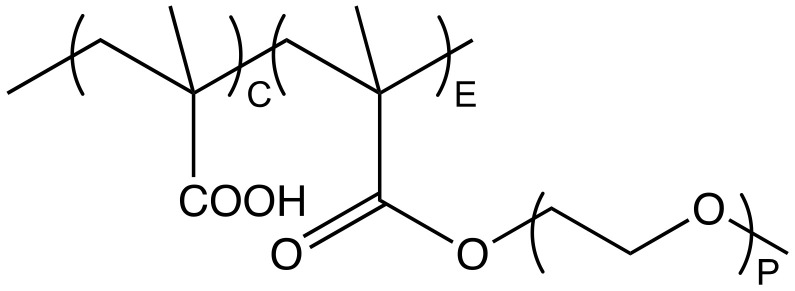
Molecular structure of a comb shaped PCE featuring a methacrylic backbone and PEG side chains with P repeating units. C notes the number of MAA units in the backbone and E refers to the number of side chain bearing backbone units.

Water-soluble polymers are widely applied as dispersing agents for aqueous particle dispersions, including gypsum [1], limestone [2], silica [3] and concrete [4]. In this paper, we focus on the specific case of comb copolymers that can be synthesized with systematic structural variations and for which structure-conformation relations were already reported in this journal [5]. More specifically, the polymers we examine contain an anionic backbone given by poly(methacrylic acid) (PMAA) and poly(ethylene glycol) (PEG) side chains. In the field of construction chemicals, these comb copolymers are often referred to as poly(carboxylate ethers) (PCEs), with a representative molecular structure shown in Figure 1.

As synthetic polymers, PCEs have to be considered as multicomponent materials. On the molecular level, they reveal an assembly of macromolecules with variations regarding backbone length, side chain length and grafting density. Consequently, a PCE cannot be completely described by a single distinct value for a structural property, but must be described by a distribution thereof, for example by giving the shape and width of the molar mass distribution (MMD) [6,7,8,9].

While the MMD does not provide the full picture of heterogeneity, it is very useful since many polymer properties, such as solubility [10] and melting point, are closely related to the molar mass. However, regardless of their applications, the performance of polymers is also conditioned by their specific chemical nature and this is particularly true for the type of dispersants discussed in this paper. Also, while the MMDs of such polymers can be approached with well-established size-exclusion chromatography (SEC) protocols, accessing chemical composition distributions (CCD) is much more challenging and to date has not been achieved for hydrophilic PCE copolymers.

As a result, although the role of molecular parameter averages has been widely studied [11,12,13,14,15,16,17,18] for PCEs, the role of their variations has received very little attention [19,20]. However, information about the dispersity in molar mass and chemical composition is essential to predict, understand and tailor the performance of dispersants.

In this paper, we therefore aim to provide a deeper insight into the molecular heterogeneity of PCEs, establishing the needed analytical methods and applying these to demonstrate that different synthesis pathways can lead to clearly different chemical heterogeneity. For this, we apply SEC with dual concentration detection to investigate the dependence of the grafting ratio on the molecular size for various PCE samples. In doing so, we use a multi-angle laser light scattering (MALLS) setup with online refractive index (RI) detection to target the MMD. Along with this, simultaneous UV detection enables us to quantify the copolymer composition, i.e., the grafting ratio of the PCEs along the elution axis. The results not only exemplify the development of methods to characterize chemical dispersity, but also establish its relevance to better understand how various synthetic routes may affect polymer performance at equivalent average composition.

The paper is divided in two parts. First, homopolymer mixtures of PMAA (precursor backbone) and PPEGMA (100% grafted backbone, poly(poly(ethylene glycol methacrylate))) of known composition are measured. These mixtures were used to evaluate the accuracy of the dual detection approach and were allowed to exclude the influence of neighboring group effects that might compromise the reliability of the dual detection results for PCE copolymers. Subsequently, in the second part of the paper, various PCE samples obtained by different synthesis pathways are subjected to dual concentration detection SEC experiments. Before this, however, we include a “background” section giving some general information first on the use of PCEs in concrete (Section 2.1) and second on PCE characterization by liquid chromatography (Section 2.2).

## 2. Background

### 2.1. PCEs in Concrete

A field that takes significant advantage of PCEs but where the influence of molecular heterogeneity has widely been neglected so far is concrete technology [19,20,21,22]. In the construction sector, PCEs belong to the broader family of chemical admixtures referred to as superplasticizers (SPs) [4,22]. Their usage has reached an estimated volume of more than 3 million tons per year (based on 30 % liquid concentration) [22,23]. A particular benefit of using PCEs is that they facilitate producing concrete with decreased environmental impact and improved rheology without compromising the final performance and strength of the building material [24].

Due to the charged carboxylate groups along the backbone, the molecules tend to adsorb to the surface of mineral phases in cement [22,25]. Upon adsorption, interparticle attractions are reduced due to steric hindrance resulting in a decreased yield stress and improved fluidity [25,26,27].

PCEs have enabled substantial performance improvements thanks to the flexibility they offer in designing molecular architecture. Various molecular designs can be realized via multiple synthesis pathways. Industrially, the most relevant one is the free radical copolymerization (FRC) of methacrylic acid (MAA), acrylic acid or maleic acid with a macromonomer (i.e., poly(ethylene glycol methacrylate), PEGMA) [4]. Alternatively, PCEs are obtained by grafting mono-hydroxylated PEG derivates onto preformed polycarboxylate backbones, such as poly(methacrylic acid) (PMAA) or poly(acrylic acid) (PAA) [4,28,29,30].

Each synthesis route includes one step of conventional free radical polymerization to either produce the precursor or to obtain the combs via copolymerization. Consequently, the resulting PCEs exhibit broad molar mass distributions. Controlled polymerization techniques, such as RAFT polymerization, can also be applied for PCE synthesis. However, due to increased costs of the RAFT process, RAFT-PCEs are of minor importance on industrial scales [28,31].

### 2.2. PCE Characterization

Investigations of heterogeneity within a PCE are often restricted to the dispersity with respect to molar mass, while chemical heterogeneity (i.e., variations in the grafting ratio) is widely neglected. However, conventional PCEs are disperse in more than one aspect, i.e., molar mass and chemical composition [5,32]. Therefore, a comprehensive characterization of all distributions is crucial to provide a better understanding of the effect of dispersity on the working mechanism of PCEs as superplasticizers [19,20].

Liquid chromatography (LC)-based techniques are powerful tools for the separation and characterization of polymers regarding molar mass distribution and chemical composition distribution (CCD). For instance, size exclusion chromatography (SEC) is a well-established method to separate polymers according to their hydrodynamic size. In the past decades, PCEs have been subject to SEC characterization. Gelardi et al. [5] showed that conventional SEC with standard calibration, although widely applied, is not suitable to obtain accurate molar mass distributions (MMD) for PCEs. Instead, they recommend using SEC in combination with structure sensitive detectors (i.e., viscosity detector, multi angle light scattering).

Alternatively, LC can be coupled with spectroscopic methods (e.g., NMR, IR, etc.) to provide insight into the CCD. A recent study used a semi-preparative approach to investigate the grafting ratio of disperse PCEs (synthesized by grafting). The samples were separated by aqueous SEC. Multiple fractions were collected manually and characterized by ^1^H-NMR. The investigation suggests a correlation between backbone length and grafting density. However, the results are preliminary and more research is needed to clarify the exact correlation [33]. Nevertheless, offline coupling of semi-preparative GPC with other spectroscopic methods is time consuming as it involves intense sample preparation. Moreover, salts added to the eluent to achieve proper chromatographic conditions can impair the resolution of spectroscopy in the second dimension and might interfere with the spectroscopic characterization.

Besides SEC, LC techniques involving interaction of the polymer with the stationary phase such as gradient chromatography (GC) or liquid chromatography under critical conditions (LCCC) provide insight into the chemical composition of PCE samples [32,34,35]. Adler et al. successfully used semi-preparative LCCC in combination with IR-detection [32,35] as well as online 2D chromatography (i.e., LCCC × SEC) [32] to investigate PCEs prepared by FRC. In the first dimension, they established critical conditions of PEG/PEO using a mixture of water and methanol. In this way, PCE molecules were separated from byproducts of the synthesis. However, these conditions did not allow to detect heterogeneity in grafting.

In the past few decades, the use of SEC with multiple concentration detectors was applied to provide quantitative information on copolymer composition and MMD [36]. Indeed, this method was successfully used for various copolymers [37,38,39,40] and blends [37,38]; however, PCEs have not been subjected to it. In this paper, we aim to elucidate information on the grafting ratio of PCEs in dependence on the molecular size using SEC in aqueous media with simultaneous RI and UV detection. With those methods then in hand, we examine how different synthesis routes can lead to different chemical heterogeneity, while targeting the same average chemical composition.

## 3. Materials and Methods

### 3.1. Applied Polymers for Dual Detection

For this study, various PMAA and PPEGMA homopolymers as well as PCE copolymers were applied. The homopolymers were obtained by free radical homopolymerization (FRP) in aqueous media. These homopolymers serve as test materials for our dual detection study (see Section 4.2). The PMAA homopolymer refers to the reference case of an ungrafted backbone (C/E = ∞) and PPEGMA corresponds to the case of a 100% grafted backbone (C/E = 0). Here, C/E refers to the numeric ratio of methacrylic acid groups to ester groups in the PCE sample.

Moreover, six methacrylic PCEs with a C/E between 1.60 and 5.0 were prepared for the second stage of this study (see Section 4.3). These PCEs were obtained by grafting a precursor backbone (PMAA; *M*w = 5300 g/mol; Ɖ = 1.4) with MPEG (*M*w = 1000 g/mol) side chains. The samples were provided by Sika AG, Zürich Switzerland. The C/E of the PCEs was determined from ^1^H-NMR spectroscopy.

To investigate the influence of the synthetic approach, three additional methacrylic PCEs were synthesized via free radical copolymerization (FRC) in aqueous media. Again, C/E was calculated from ^1^H-NMR spectroscopy. More details about synthesis and NMR evaluation can be found in Appendix A, Appendix B and Appendix C. Information on the molecular characteristics of all polymers is summarized in Table 1.

The numeric ratio between the comonomers C and E (see Figure 1) can be used to calculate the weight fraction of each component ($\omega_{C}$ and $\omega_{E}$) according to Equation (1), where *M_C_* is the molar mass of MAA and *M_E_* is the number-average molar mass of the macromonomer (PEGMA).

(1) $$\omega_{C}=\frac{C\cdot M_{C}}{C\cdot M_{C}+E\cdot M_{E}}$$

where

(2) $$\omega_{E}=1-\omega_{C}$$

### 3.2. Size Exclusion Chromatography

SEC analysis was performed on an Agilent 1260 Infinity system (Agilent Technologies, Santa Clara, CA, USA) equipped with a RI detector (Agilent Technologies, G1362A) a diode array detector (Agilent Technologies, G1315D, operated at λ = 220 nm) and a MALLS detector (SLD7100, PSS Polymer Standards Service, Mainz, Germany). A series of three PSS Suprema columns (individual dimensions 0.8 cm × 30 cm, particle size 10 µm) of different pore sizes (30, 1000, and 1000 Å) were used. The combination of MALLS and online RI was used for measuring molar mass distributions. The mobile phase (0.1 M NaCl aqueous solution, pH 10 adjusted by addition of 10 M NaOH) was pumped with a flow rate of 1 mL/min. An alkaline pH of the mobile phase is required to achieve complete deprotonation of the carboxylate groups. Moreover, the addition of salt is essential to shield interactions between the solute and the stationary phase. Notably, buffers such as Na_2_HPO_4_ or Na-acetate, which are frequently used in SEC of anionic polyelectrolytes, are not suitable for dual concentration detection of PCEs due to their UV cutoff. More information on this issue can be found in Appendix D.

The sample concentrations ranged between 1.5 and 3.0 mg/mL. For this, an adequate amount of polymer was dissolved in the eluent. For each analysis, a volume between 50 and 100 µL of polymer solution was injected. Data analysis was carried out using PSS WinGPC Software (Polymer Standards Service, Mainz, Germany).

MALLS with online RI detection allows to determine absolute MMDs of polymers. In the case of chemically disperse samples such as PCE copolymers, the refractive index increment might be different for each eluting copolymer fraction. While dual detection SEC capitalizes on this to gather information on the copolymer composition, these variations cause problems when it comes to determination of MMDs due to imprecise determination of eluting polymer concentrations [41]. However, MALLS/RI was proven to give a good estimate of the MMD for PCEs [5,42]. The corresponding weight-average molar mass, *M*w, and dispersity index, Ɖ, are presented in Table 1.

The calibration of the MALLS detector was done using a monodisperse pullulan sample (*M*w 110,000 g/mol, Ɖ = 1.12) that does not show angular dependence in scattering. The same sample was used to determine the detector constant of the RI detector as well as inter-detector delays.

### 3.3. Response Factor Determination for Dual Concentration Detection

As already mentioned above, conventional SEC data processing using a single concentration detector does not give access to the comonomer composition of a copolymer. In order to quantify the amount of comonomers, the same number of independent concentration detector signals as number of comonomers contained in the sample is needed [6,37]. With the type of PCEs considered in this study being binary copolymers, two signals (e.g., RI and UV) are needed to calculate the composition distribution and the overall bulk composition.

**Figure 2:**
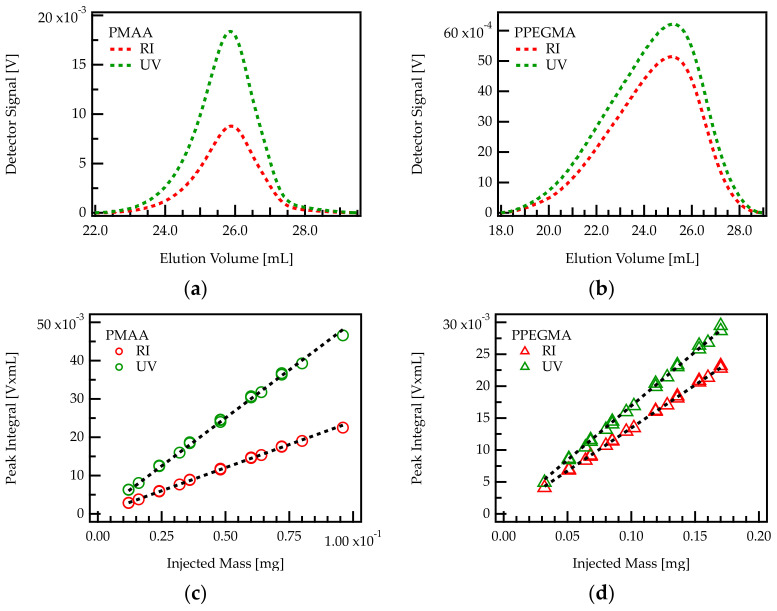
Above: Chromatogram of PMAA (**a**) and PPEGMA (**b**) homopolymers. These correspond, respectively to C/E values of ∞ and 0. The signals from RI and UV detector are plotted against the elution volume. Below: Determination of response factors in RI and UV detection of PMAA (**c**) and PPEGMA (**d**) by linear regression. The results of the linear regressions are shown in Table 2.

The DAD detector was operated at λ = 220 nm, where both the carboxylic acid groups as well as the ester groups show an absorption. Thus, both concentration detectors are able to detect both comonomers of the PCE. Notably, UV absorption is only due to the carbonyl groups in the backbone since the PEG side chains do not absorb at 220 nm. The chromatograms (RI and UV signal) for PMAA and PPEGMA (corresponding to C/E = ∞ and C/E = 0, respectively) are plotted in Figure 2a,b. Absorbance spectra of the mobile phase and an explanation of why the DAD detector was operated at 220 nm can be found in Appendix D.

A series of PMAA and PPEGMA samples with exact concentrations were injected into the chromatography setup. RI and UV signals were integrated over the eluting peaks and the peak areas were plotted against the injected mass. The integrals of both signals turn out to be proportional to the injected mass of homopolymer, as shown in Figure 2c,d.

The slopes of peak area versus injected mass provide the response factor for each homopolymer–detector combination. The response factor of PMAA in RI detection will be referred to as $k_{C}^{RI}$ and in UV detection $k_{C}^{UV}$. The response factors of PPEGMA are termed analogously $k_{E}^{RI}$ and $k_{E}^{UV}$.

Notably, the determination of response factors for polyelectrolytes is delicate. The method described above is suggested in literature [43,44] to ensure an equilibrium distribution of counter ions in the vicinity of the polymer and the bulk solution.

## 4. Results and Discussion

### 4.1. Evaluation of Dual Concentration Results

The signals of both detectors, *S^RI^* and *S^UV^*, are concentration sensitive and depend on the chemical nature of the sample. Hence, for copolymers, the signal from the *i*th slice of the chromatogram is given by the signal contribution of each component [6,37,40]:

(3) $$S_{i}^{RI}=c_{i,P}\cdot \left[\omega_{i,C}\cdot k_{C}^{RI}+\omega_{i,E}\cdot k_{E}^{RI}\right]$$

(4) $$S_{i}^{UV}=c_{i,P}\cdot \left[\omega_{i,C}\cdot k_{C}^{UV}+\omega_{i,E}\cdot k_{E}^{UV}\right]$$

where $c_{i,P}$ refers to the concentration of the polymer in the *i*th fraction of the chromatogram, while $\omega_{i,C}$ and $\omega_{i,E}$ are the weight fractions of comonomers in that *i*th slice. Here, the indices C and E refer to MAA and PEGMA as comonomers. $k^{RI}$ and $k^{UV}$ are the response factors of the homopolymers (Table 2).

Additionally, we find that:

(5) $$\omega_{E}=1-\omega_{C}$$

From the above, for each slice, we have a system of three equations for the three unknowns ($c_{i,P}$, $\omega_{i,C}$ and $\omega_{i,E}$) that can be solved to yield information on the concentration and composition of the eluting fraction as described in literature [37,41]. Equations (3)–(5) can be used to follow the sample composition along the elution axis by considering the signal ratio at time *i*. Moreover, the overall composition of a sample can be calculated by considering the integral over the complete RI and UV peak.

### 4.2. Mixtures of PMAA and PPEGMA Homopolymers

In contrast to most applications of SEC with dual concentration detection, both comonomers of our PCEs give rise to a response in both detectors, thereby challenging analysis. In order to find out if the method is capable of correctly quantifying relative amounts of C and E, we decided to analyze homopolymer mixtures before changing to PCEs. For this purpose, a series of 20 homopolymer (PMAA and PPEGMA) mixtures of known compositions were prepared and analyzed. The composition of the mixtures is given according to their weight fraction of PMAA. For instance, $\omega_{C}^{weight}=$ 0.05 corresponds to a mixture with 5 wt% of PMAA and 95 wt% of PPEGMA.

**Figure 3:**
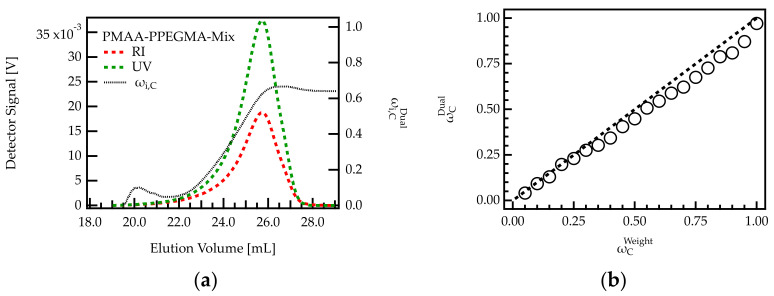
Analysis of homopolymer mixtures using dual detection. (**a**) Chromatogram of a sample with $\omega_{C}^{Weight}=$ 0.7. The composition of the eluting fractions was monitored. (**b**) Comparison of $\omega_{C}^{Weight}=$ 0.7 with $\omega_{C}^{Dual}=$ 0.7 for 20 homopolymer mixtures of different PMAA and PPEGMA content.

An example of a chromatogram obtained for such mixtures is shown in Figure 3a for the mixture with $\omega_{C}^{weight}=$ 0.7. The sample elutes between 18 and 28 mL, which agrees with the elution volume of the homopolymers (Figure 2a,b).

The composition of the eluting species was calculated across the peak using RI and UV detection. Upon the onset of elution (18–22 mL), both concentration signals are weak. Hence, the calculated composition, $\omega_{i,C}^{Dual}$ cannot be considered precise. For volumes higher than 22 mL, $\omega_{i,C}^{Dual}$ is lower than 0.5, indicating that mainly PPEGMA is eluting from the column. With increasing elution volume, $\omega_{i,C}^{Dual}$ increases until a value of 0.63. The increase in $\omega_{i,C}^{Dual}$ with elution volume agrees with the elution profile of the homopolymers. Due to its larger hydrodynamic volume, PPEGMA starts eluting earlier than PMAA (see Figure 2a,b).

To obtain information on the overall composition of the sample, the RI and UV signals shown in Figure 3a were integrated and $\omega_{C}^{Dual}$ of the complete mixture was calculated as described in Section 4.1. The calculated value of $\omega_{C}^{Dual}=$ 0.62 is lower than the expected fraction of 0.70 for the specific sample shown in Figure 3a.

For a more detailed analysis of the deviation, the integration was carried out for all homopolymer mixtures. The determined weight fractions of PMAA were plotted against the weighted amounts. Figure 3b shows that all data points follow the trend of a bisecting line, indicating that $\omega_{C}^{Weight}$ and $\omega_{C}^{Dual}$ are in good agreement. For samples with low PMAA content (high PPEGMA), the data points show an almost perfect match. However, for samples with high PMAA content (low PPEGMA), $\omega_{C}^{Dual}$ is systematically smaller than $\omega_{C}^{Weight}$. This deviation may be due to the lower UV response factor of PPEGMA compared to PMAA (Table 2). For low concentrations of PPEGMA, it only has a small contribution to the overall UV signal. If the signal contribution is below the detection limit, the composition of the mixture will not be captured correctly. Notably, this deviation is not expected to impact PCE characterization of the samples in the present investigation as all relevant PCEs have a PMAA content lower than 0.35 (Table 1).

### 4.3. Dual Detection SEC of PCEs

#### 4.3.1. Comparison with ^1^H-NMR

**Figure 4:**
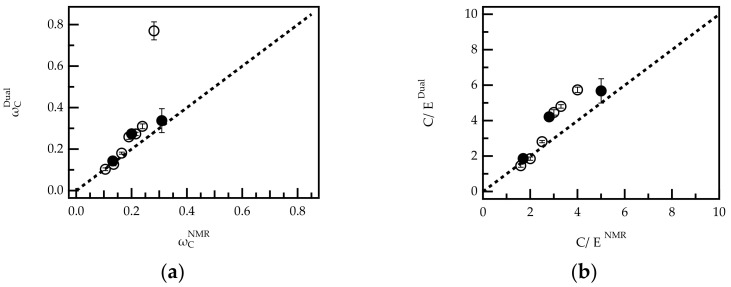
Comparison of copolymer composition in G-PCEs (O) and FRC-PCEs (l) calculated from ^1^H-NMR data and dual concentration detection SEC expressed as weight fraction of comonomer C (*ω_C_*) (**a**) and C/E ratio (**b**). The results for sample G-PCE-5.0 are excluded from graph (**b**).

The above results show that dual detection can quantify amounts of monomers C and E in mixtures of pure reference compounds (homopolymers). To verify whether this also applies to the quantification of comonomers in PCEs, the method was compared to ^1^H-NMR data. For this, all PCEs shown in Table 1 were investigated by dual detection SEC. The overall weight fraction of comonomer C ($\omega_{C}^{Dual}$) in each sample was calculated according to Equations (3)–(5) after integration over the whole RI and UV signal. The obtained weight fractions were compared to the average composition calculated from ^1^H-NMR spectra ($\omega_{C}^{NMR}$) (Figure 4).

Figure 4 shows a plot of $\omega_{C}^{Dual}$ vs. $\omega_{C}^{NMR}$ for G-PCEs and FRC-PCEs and a bisecting line (dashed line). In the case of perfect agreement between both analytical techniques, the data points are expected to fall on this line. Indeed, for PCEs with a high grafting ratio (low weight fraction of $\omega_{C}$), the analysis by NMR and dual detection are in good agreement. For $\omega_{C}^{NMR}$ > 0.2, the weight fractions determined from dual detection are slightly higher than the corresponding NMR values, but still a clear trend can be observed. Only for G-PCE-5.0 ($\omega_{C}^{NMR}$ = 0.281), NMR and dual detection analysis deviate significantly. The reasons for the deviation were not further investigated. However, G-PCE-5.0 was excluded from further dual detection analysis in the second part of the paper. Notably, FRC-PCE-5.0 ($\omega_{C}^{NMR}$ = 0.281), which has a very similar molar composition to G-PCE-5.0, follows the trend of the bisecting line.

The agreement between $\omega_{C}^{Dual}$ and $\omega_{C}^{NMR}$ proves that the comonomer content in PCEs can precisely be quantified using dual detection SEC without being compromised by neighboring group effects. This result brings us to the next part of this paper where we will focus on tracing the PCE composition along the elution axis of the chromatogram.

#### 4.3.2. On the Homogeneity of PCEs produced by Esterification

Grafting of precursor backbones is a widely used technique for the preparation of PCE model structures for research purpose. It is often claimed that grafting leads to a homogenous distribution of side chains along the backbone. Moreover, the independence of the grafting ratio from backbone length it commonly assumed. However, lately some doubts have been raised about whether the dispersity of the backbone length in the precursor P(M)AA might impact the grafting ratio [33].

**Figure 5:**
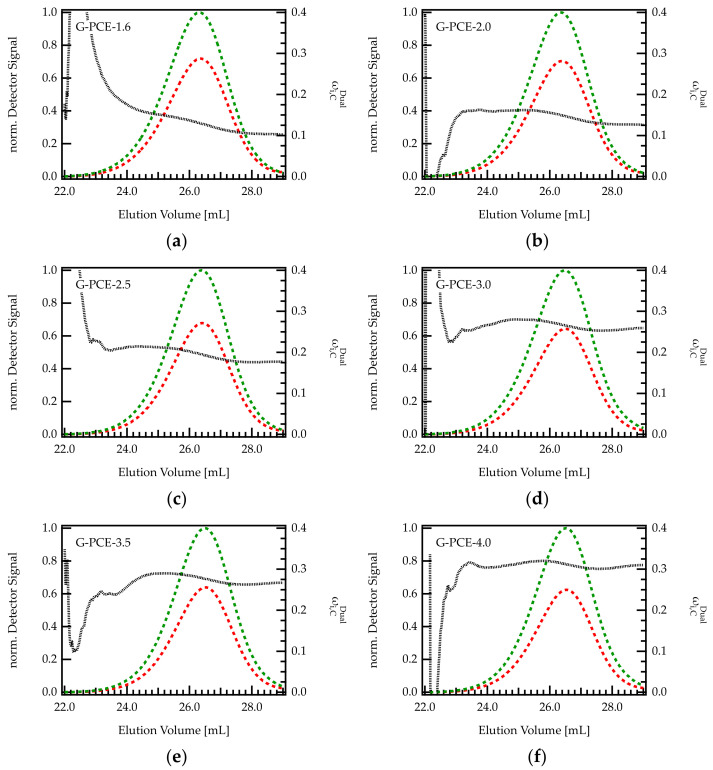
(**a**–**f**) Chromatograms of various G-PCE samples with different C/E ratios. The green curve is the UV signal, and the red curve corresponds to the RI signal. The signals were normalized with regard to the maximum of the UV peak. The dashed black line indicates the composition of the eluting species monitored by dual detection.

Dual detection SEC offers the opportunity to monitor the composition of the grafting ratio along the elution peak in a chromatogram Figure 5 shows the chromatogram (RI and UV signal) and the content of C ($\omega_{i,C}^{Dual})$ within six different G-PCE samples.

For all PCEs, $\omega_{i,C}^{Dual}$ seems to be rather constant, with only a slight decrease in $\omega_{i,C}^{Dual}$ with increasing elution volume. Thus, early eluting fractions (larger hydrodynamic volume) contain a slightly higher weight fraction of C than late eluting fractions. This behavior is more pronounced for PCEs with low C/E ratio (high grafting degree). For instance, for G-PCE-1.6, $\omega_{i,C}^{Dual}$ decreases by 6.6 wt%, between 24 and 28 mL. This corresponds to a change in C/E from 2.7 to 1.5, which is substantial in regard to PCE performance as superplasticizers. In contrast, $\omega_{i,C}^{Dual}$ of G-PCE-4.0 is almost constant at 30.3 wt% throughout the chromatogram. It has to be mentioned that the calculated values of $\omega_{i,C}^{Dual}$ are fluctuating at peak start and end. These uncertainties are due to low concentrations and corresponding weak detector signals of the eluting species in this area.

According to Figure 5, it appears that PCEs with a smaller hydrodynamic size (early elution) feature a slightly higher content of C than bigger molecules. It is conceivable that such differences in the grafting ratio are related to the backbone length of the precursor. Indeed, the backbone of the G-PCEs features a *M*w of 5300 g/mol, with dispersity index Ɖ = 1.4 meaning that the molar mass ranges approximately between 100 and 20,000 g/mol. The impact of backbone length on grafting ratio is further discussed in the next section.

#### 4.3.3. Impact of Backbone Length

**Figure 6:**
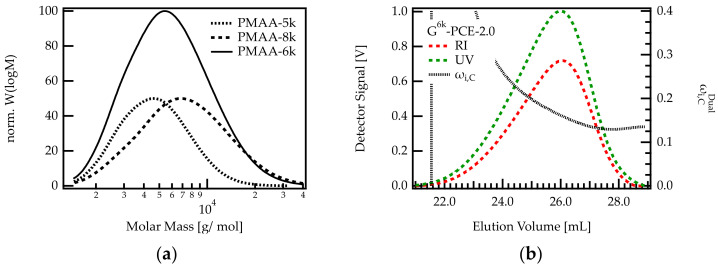
(**a**) Molar mass distributions of PMAA. PMAA-5k was used for the G-PCEs shown in Table 1. The molar mass range of this PCE was increased by adding PMAA-8k. The resulting backbone mix contains PMAA molecules with molar masses between approximately 100 and 40,000 g/mol. (**b**) Dual detection SEC analysis of G6k-PCE-2.0.

In order to verify if the backbone length impacts the C/E ratio of the PCE, a G-PCE was produced by grafting a mixture of two backbones with different molar masses. This mixture included the previously used PMAA-5k backbone and a larger one: PMAA-8k (*M*w 8100; Ɖ = 1.5) in proportions of 1:1 by weight. The mix is referred to as PMAA-6k, whereby its average molar mass is *M*w 6400 g/mol with Ɖ = 1.4. With this mix, the molar mass range of the grafted PCE was extended to higher molar masses (up to 40,000 g/mol). The molar mass distributions of all PMAAs are shown in Figure 6.

The results in Figure 5 show that the heterogeneity of the C/E ratio is more pronounced for G-PCEs with a C/E ratio below approx. 2.5. Therefore, a C/E of 2 was targeted when grafting sidechains onto PMAA-6k. The resulting G-PCE is termed G^6k^-PCE-2.0.

As can be seen in Figure 6b, the dual detection analysis of G^6k^-PCE-2.0 shows that $\omega_{i,C}^{Dual}$ decreases along the elution axis. Early eluting PCE fractions feature a higher methacrylic acid content compared to later eluting species. Between the onset and end of the elution peak, the weight fraction of C decreases by more than 13 wt%. This corresponds to a change in C/E from 3.9 to 1.7. This decrease is significantly stronger than for G-PCE-2.0, where the weight fraction of C is reduced by roughly 3.1 wt%, meaning that the C/E varies between 2.4 and 1.9. Comparing G-PCE-2.0 and G^6k^-PCE-2.0 confirms that the length of the precursor backbone impacts the grafting density of the PCE. It appears that small backbones tend to feature a higher grafting degree than longer backbones.

#### 4.3.4. PCEs from Free Radical Copolymerization

**Figure 7:**
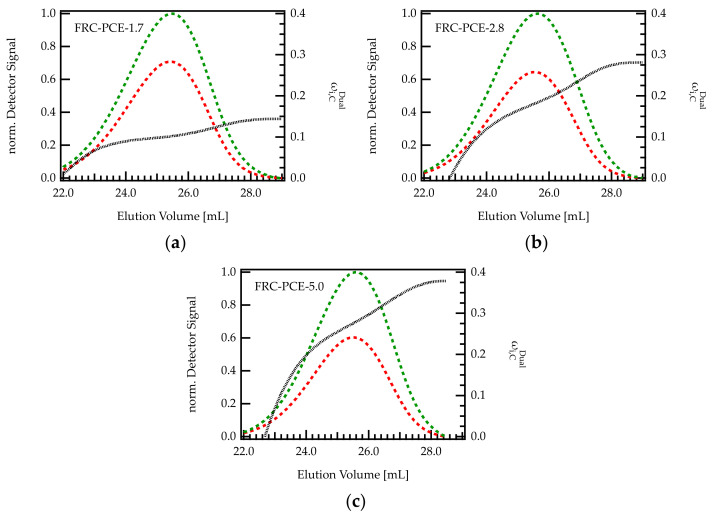
(**a**–**c**) Chromatogram of various FRC-PCE samples with different C/E ratio. The green curve is the UV signal, and the red curve corresponds to the RI signal. The signals were normalized with regard to the maximum of the UV peak. The dashed black line indicates the composition of the eluting species monitored by dual detection.

On industrial scales, PCEs are most often obtained by free radical copolymerization. To reflect this mode of production, three different FRC-PCEs were characterized with dual detection SEC in order to investigate the methacrylic acid content across the elution peak. As can be seen from Figure 7, the $\omega_{i,C}^{Dual}$ increases with increasing elution volume, indicating that larger molecules (early elution) are more extensively grafted than smaller molecules (late elution). Thus, the trend is reversed compared to G-PCEs. Notably, the differences in $\omega_{i,C}^{Dual}$ between the onset and end of the elution peak are more pronounced for PCEs with high C/E ratio.

Generally speaking, it is difficult to ascribe the dispersity of the grafting density to one particular impact factor during FRC synthesis. The molecular architecture of FRC-PCEs depends on many factors among them the reactivity ratio [29,45] of the comonomers, the monomer feed during synthesis, but also the choice of chain transfer agent or possible side reactions such as radical transfer and termination have to be considered. While the topic deserves further investigation, these results underpin the existence of this inhomogeneity, representing an additional factor that should be considered when studying the working mechanisms of such compounds or seeking to improve their performance.

## 5. Conclusions

It has been shown that aqueous size exclusion chromatography with dual concentration detection is a suitable tool to characterize the grafting ratio of poly(carboxylate ethers). It was revealed that the synthesis pathway has a significant impact on the variation of grafting density with molar mass.

Polymer analogous esterification revealed a weak correlation between the backbone length and grafting ratio. It appeared that smaller backbones carry more side chains than larger ones. Variations in the grafting ratio are more expressed when a high grafting density is targeted during synthesis. This result is of particular interest for admixture research where G-PCEs are often used as model structures. It is often assumed that within a G-PCE sample, all backbones feature the same grafting ratio and are only disperse with regard to the backbone length. However, this study proves that PCEs from esterification are disperse in a second dimension, i.e., comonomer content. For the synthesis of optimal model structures where the chemical heterogeneity can be neglected, we suggest using narrowly distributed backbones as precursor.

Having said this, PCEs from free radical copolymerization show much stronger variation in the grafting ratio with molar mass. While larger molecules are highly grafted, smaller molecules have a higher methacrylic acid content. Consequently, the correlation between molar mass and grafting ratio is reversed for FRC-PCEs compared to G-PCEs.

In literature, it has been shown that the charge density significantly impacts the affinity of PCE molecules for adsorption on the cement surface [16,19,46]. Molecules with high C/E ratio were found to preferentially adsorb over molecules with shorter backbones and high grafting degree. PCEs with decreased efficiency demand higher dosages to achieve the same workability of the concrete. Hence, the suggested dual detection method offers a tool to identify fractions within a PCE sample that are potentially less effective regarding their plasticizing ability. This suggests that new strategies to adapt the synthesis conditions may enable the production of more efficient PCEs with tailormade molecular structure.

Liquid chromatography is a well-established tool to characterize polymers. With regard to PCE analysis, the simple addition of a second concentration detector to a standard SEC setup can provide new insights into the chemical dispersity of PCEs. Revealing their chemical heterogeneity gives access to a better understanding of their molecular heterogeneity.

While our paper is focused on PCEs, it is suggested that the dual detection method in aqueous media can also be applied to other types of water-soluble copolymer with different components and architectures. For this purpose, only suitable detector response factors for the comonomers need to be established.

The most important characteristic that conditions the suitability of dual concentration detection is a sufficiently different response of the comonomers in at least one detector. When neighboring group effects can be excluded, the method is equally suitable for random copolymers, gradient and (multi)block structures.

Besides PCEs, further copolymers containing (meth)acrylic acid or maleic acid as comonomer can be characterized by dual concentration detection. Moreover, acrylamide, N-isopropyl acrylamide vinyl acetate or lactic acid containing copolymers can potentially be subjected to this method, too.

All in all, dual detection SEC in aqueous media is a versatile and promising tool to understand molecular heterogeneity in water-soluble copolymers. The collected information is of particular interest to comprehend the structure–performance relations of functional polymers, as exemplified here in the case of superplasticizers extensively used in cementitious materials.

## Figures and Tables

**Table 1:** Average molecular characteristics of applied PCEs. The numeric ratio between the comonomers (C/E) was obtained from ^1^H-NMR spectroscopy. The corresponding weight fractions of component C and E were calculated according to Equation (1). P gives the number of repeating units in the PEG side chain. *M*w and the dispersity index, Ɖ, were measured via SEC using online refractive index and MALLS detection.

Name	Synthesis	C/E (NMR)	$\omega_{C}$	$\omega_{E}$	P	M_w_	Ɖ
^1^ PPEGMA	FRP	0.00	0.00	1.00	22	339.6	3.2
^1^ PMAA	FRP	∞	1.00	0.00	-	5.3	1.4
^1^ G-PCE-1.6	Grafting of Precursor Backbone	1.6	0.112	0.888	22	25.4	1.6
^1^ G-PCE-2.0		2.0	0.135	0.865	22	22.5	1.5
^1^ G-PCE-2.5		2.5	0.164	0.836	22	18.0	1.5
^1^ G-PCE-3.0		3.0	0.190	0.810	22	17.7	1.5
^1^ G-PCE-3.3		3.3	0.203	0.797	22	15.1	1.5
^1^ G-PCE-4.0		4.0	0.239	0.761	22	13.2	1.6
^1^ G-PCE-5.0		5.0	0.281	0.719	22	16.6	1.8
FRC-PCE-1.7	FRC	1.7	0.132	0.868	19	67.1	1.7
FRC-PCE-2.8	FRC	2.8	0.200	0.800	19	51.6	1.9
FRC-PCE-5.0	FRC	5.0	0.309	0.691	19	23.4	1.7

^1^ Samples were kindly provided by Sika AG, Switzerland.

**Table 2:** Response factors for PMAA and PPEGMA. The UV response factors were determined for a detection wavelength of 220 nm.

Sample	*k^RI^* [(V × L)/g]	*k^UV^* [(V × L)/g]
PMAA	0.241	0.501
PPEGMA	0.135	0.169

**Figure A1:**
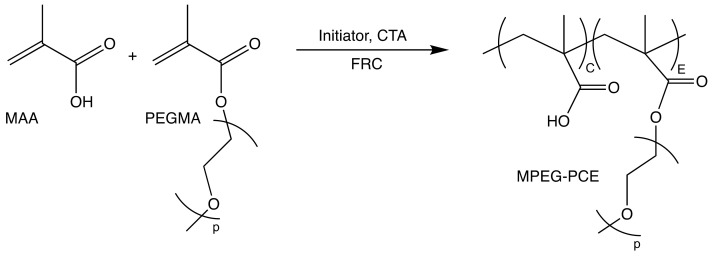
Free radical copolymerization route for the synthesis of MPEG-type PCEs.

**Figure A2:**
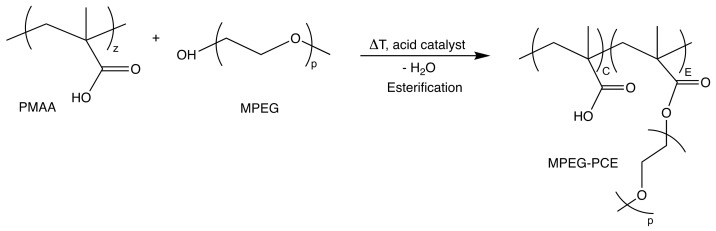
Grafting process (polymer analogous esterification) for the synthesis of G-PCEs.

**Figure A3:**
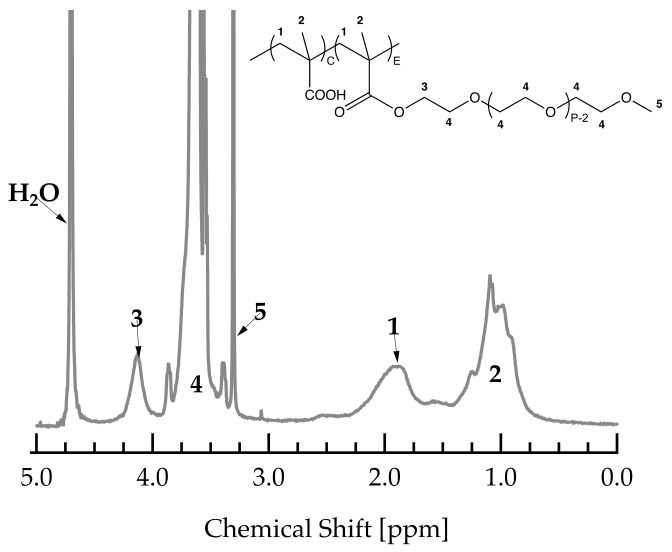
^1^H-NMR spectrum of a PCE with PMAA backbone and MPEG side chains.

**Figure A4:**
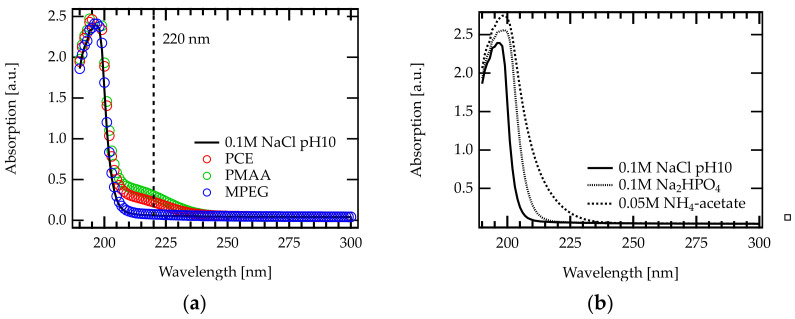
UV-Vis absorption spectra recorded between 190 and 300 nm: (**a**) various polymers dissolved in 0.1 M NaCl at pH 10. The UV cutoff of this solvent is located at approximately 210 nm. For PCE and PMAA solution, an absorption peak was found at 220 nm. MPEG does not show an absorption peak in the recorded range. (**b**) Different aqueous salt/buffer solutions that are frequently used in SEC analysis of PCEs. The addition of salt/buffer affects the cutoff wavelength.

## Data Availability

Not applicable.

## List of Math Symbols and Abbreviations

List of Abbreviations.

**Abbreviation**	**Description**
CCD	chemical composition distribution
Ɖ	dispersity index
FRC	free radical copolymerization
GC	gradient chromatography
IR	infrared
LC	liquid chromatography
LCCC	liquid chromatography at critical conditions
MALLS	multi angle laser light scattering
(M)PEG	(methoxy) poly(ethylene glycol)
MMD	molar mass distribution
M_n_	number-averaged molar mass
M_w_	weight-averaged molar mass
NMR	nuclear magnetic resonance
PCE	poly(carboxylate ether)
PEGMA	poly(ethylene glycol) methyl ether methacrylate
PMAA	poly(methacrylic acid)
PPEGMA	poly(poly(ethylene glycol) methyl ether methacrylate)
RI	refractive index
SEC	size exclusion chromatography
UV	ultraviolet

List of Math Symbols.

**Symbol**	**Description**
C	comonomer C (=MAA) (used in math symbol)
E	comonomer E (=PEGMA) (used in math symbol)
$\omega_{C}$	weight fraction of component C
$\omega_{E}$	weight fraction of component E
$M_{C}$	Molar mass of of C
$M_{E}$	Molar mass of of E
λ	detection wavelength in UV detection.
$k_{C}^{RI}$	response factor of C in RI detection
$k_{C}^{UV}$	response factor of C in UV detection
$k_{E}^{RI}$	response factor of E in RI detection
$k_{E}^{UV}$	response factor of E in UV detection
$S_{i}^{RI}$	RI signal for *i*th slice of the chromatogram
$S_{i}^{UV}$	UV signal for *i*th slice of the chromatogram
$c_{i,P}$	concentration PCE for *i*th slice of the chromatogram
$\omega_{i,C}$	weight fraction of C in *i*th slice of the chromatogram
$\omega_{i,E}$	weight fraction of E in *i*th slice of the chromatogram
$\omega_{C}^{Dual}$	overall weight fraction of C determined by dual concentration detection
$\omega_{C}^{NMR}$	overall weight fraction of C determined by ^1^H-NMR

## Appendix A

### Free Radical Copolymerization of PCEs

FRC-PCEs with different grafting densities were obtained from radical copolymerization of methacrylic acid (MAA; contains 250 ppm 4-methoxyphenol (MEHQ) as the inhibitor; >99%) and poly(ethylene glycol) methyl ether methacrylate (PEGMA M_n_ 950 g/mol, with 100 ppm MEHQ as the inhibitor; Sigma Aldrich, Buchs, Switzerland) in aqueous media. As a chain regulator, sodium 3-mercaptopropionic acid (MPA; for synthesis; Sigma Aldrich) was used, and potassium persulfate (K_2_S_2_O_8_; ACS reagent grade >99.0%, Sigma Aldrich) was applied as a radical initiator. Water was purified by a Millipore Milli-Q filtration system from Merck, Zug, Switzerland (TOC < 2 ppb). The exact amounts of educts are listed in Table A1.

**Table A1:** Applied chemicals for free radical copolymerization of PCEs with different grafting degree.

Name		Solution A				Solution B		Reactor
	$\frac{\left[MAA\right]}{\left[PEGMA\right]}$	MAA [mol]	PEGMA [mol]	Water [mL]	MPA [mol]	K_2_S_2_O_8_ [mol]	Water [mL]	Water [mL]
FRC-PCE-1.7	1.5	0.0233	0.0155	4.4	0.0013	0.0004	18.0	11.2
FRC-PCE-2.8	3.0	0.0291	0.0097	3.0	0.0013	0.0004	12.2	8.3
FRC-PCE-5.0	5.0	0.0581	0.0116	4.0	0.0023	0.0008	16.1	10.8

Prior to synthesis two solutions are prepared. Solution A contains the comonomers (MAA and PEGMA-19), ultrapure water, and mercaptopropionic acid. Solution B contains potassium persulfate dissolved in ultrapure water. A five-neck round bottom flask (total volume 100 mL) was filled with water and was subsequently heated to 80 °C while being flushed with nitrogen to remove oxygen from the solution. The solution was stirred with an overhead stirrer (IKA^®^ Eurostar power control visc). The nitrogen bubbling was continued during the whole synthesis process. After 20 min of heating and degassing, solutions A and B were added to the reactor. For this purpose, a peristaltic pump (Ismatec ISM831C) was used to maintain a constant flow rate. Solution A was pumped with a rate of 0.225 mL/min and B was added at 0.141 mL/min. After the addition of both solutions was finalized, the polymer solution was stirred for one more hour at 80 °C. During the reaction, the viscosity of the solution increased significantly. After cooling to room temperature, a viscous polymer solution was obtained which was purified by dialysis. Subsequently, the C/E ratio of the PCEs was determined from ^1^H-NMR measurements.

## Appendix B

### Grafting of a Precursor Backbone

G-PCEs were obtained via grafting of a precursor backbone (PMAA-5k, M_w_ 5300 g/mol, Đ = 1.4) with mono-hydroxylated MPEG (M_w_ 1000 g/mol, BASF SE) in a polymer-analogous esterification process. The process can be regarded as an acid-catalyzed Fischer-esterification. The reaction was carried out in melt. High temperature and low vacuum are needed to yield high conversions. All G-PCEs were provided by Sika AG, Zürich, Switzerland. Details about the synthesis procedure are not disclosed. The C/E ratio was calculated from ^1^H-NMR measurements.

## Appendix C

### C/E Ratio from ^1^H-NMR Measurements

The average C/E ratio was determined by ^1^H-NMR measurements. All measurements were carried out on a Bruker 300 MHz spectrometer using D_2_O as a solvent. Figure A3 shows a typical spectrum of a PCE with a PMAA backbone and MPEG side chains including peak assignments.

C/E can be calculated using the protons of the backbone (five protons in peak 1 and 2) and those of the terminal methyl-group of the side chain (three protons in peak 5). To this end, peaks 1, 2 and 5 were integrated and normalized by the corresponding number of protons. Subsequently, the C/E can be calculated as follows:

(A1) $$C/E=\frac{I_{1}+I_{2}-I_{5}}{I_{5}}$$

where $I_{1}$, $I_{2}$ and $I_{5}$ represent the normalized integrals of peaks 1, 2 and 5, as shown and assigned in Figure A3.

## Appendix D

### Choice of Mobile Phase and Detection Wavelength

In the past, dual concentration detection SEC of copolymers was carried out mostly in organic solvents [37,38,39,40]. Many organic solvents show their UV cutoff for wavelengths longer than 220 nm [47]. This does not allow to detect carbonyl groups, which feature their absorption maximum approximately at this wavelength (Figure A4a). Notably, the ether groups of the MPEG side chains do not absorb at 220 nm (Figure A4a, blue).

Figure A4b shows the UV-Vis spectra of several aqueous buffer solutions (i.e., Na_2_HPO_4,_ and NH_4_-acetate) that are frequently used as mobile phases for SEC analysis of PCEs [33]. These buffers feature a UV cutoff ≥ 220 nm, hence the absorption of carbonyl groups cannot be detected.

In contrast, the addition of NaCl (and NaOH) results in a UV cutoff at 210 nm allowing to detect the carboxylate and ester peak. According to the best of our knowledge, the present study is the first to perform dual concentration detection SEC in aqueous media.

All absorption spectra were measured in the wavelength range between 190 and 300 nm (wavelength steps of 1 nm) using a Lambda 650 UV-Vis spectrophotometer from Perkin Elmer. The solutions were filled into a quartz cuvette (Hellma^®^, QS Quartz Glass High performance, path length 10 mm). All spectra were recorded using air as a reference.

In addition to the UV cutoff of the mobile phase, any kind of functional group and end group modification must be considered when developing a dual concentration detection protocol. When the end group and repeating unit show an overlap in their absorption spectra, dual concentration detection might not allow us to correctly quantify the copolymer composition, when the detection wavelength is located within the overlap region. For the PCE samples applied in this study, this is not of concern. However, we want to point out this potential issue for readers who may be interested in applying dual concentration detection to other types of copolymers.

Generally speaking, the detection wavelength has to be selected appropriately that the end group contribution compared to absorption of the repeating units is negligible. In particular for high molar mass polymers, this requirement is often fulfilled as the number of repeating units is much higher than the number of end groups.

However, strongly absorbing end groups, such as thiocarbonylthio-based compounds that are frequently applied in RAFT polymerization, might interfere with the UV detection of the comonomers [48].
